# Effect of exogenous progesterone treatment on ovarian steroid hormones and oxidant and antioxidant biomarkers during peak and low breeding seasons in dromedary she-camel

**DOI:** 10.14202/vetworld.2019.542-550

**Published:** 2019-04-17

**Authors:** Amal M. Abo El-Maaty, Ragab H. Mohamed, Heba F. Hozyen, Adel M. El-Kattan, Mona A. Mahmoud, Amal H. Ali

**Affiliations:** 1 Department of Animal Reproduction and Artificial Insemination, Veterinary Division, National Research Center, Dokki, Giza, Egypt; 2 Department of Theriogenology, Faculty of Veterinary Medicine, Aswan University, Egypt; 3 Department of Animal Health, Desert Research Center, Cairo, Egypt

**Keywords:** breeding season, camel, controlled internal drug release, ovarian hormones, oxidant/antioxidant biomarkers

## Abstract

**Background:**

Research about the effects of progesterone (P_4_) and the relationship of P_4_ to oxidative stress has been achieved in ruminants but not enough in camels.

**Aim:**

This study evaluated the effect of exogenous P_4_ hormone using CIDR for 7 days on blood concentrations of steroid hormones and oxidative status of dromedary she-camels during peak and low breeding seasons.

**Materials and Methods:**

The present work was conducted on ten dark dromedary she-camels which were synchronized using a controlled internal drug release (CIDR) for 7 days as a reproductive management tool during peak breeding (November-April) and low breeding season (May-October). The blood samples were collected each other day from CIDR insertion until the end of experiment 5 days after the removal of CIDR. Camels were examined for P_4_, estradiol (E_2_), and testosterone (T) as well as malondialdehyde (MDA) as indicator of lipid peroxidation and nitric oxide, superoxide dismutase (SOD), and glutathione-S-transferase as antioxidant markers.

**Results:**

Results revealed that P_4_ was higher during peak breeding season than low breeding season. While the levels of P_4_ increased during CIDR insertion and declined at CIDR removal and thereafter during breeding season, its concentrations declined after CIDR application during the non-breeding season. On the other hand, blood E_2_ and testosterone levels decreased after CIDR insertion in both high and low breeding seasons with higher serum E_2_ concentrations during the peak than the low breeding season. MDA concentrations and SOD activities were significantly (p<0.05) high on day 3 after CIDR insertion during the breeding and non-breeding seasons. During both the seasons, GSH levels decreased after CIDR removal in camels. However, MDA was lower during non-breeding season than high breeding season with no seasonal effect on SOD activity.

**Conclusion:**

Exogenous P_4_ treatment through CIDR in dromedary camels could be more efficient during breeding season than non-breeding season, and effects on circulating oxidant/antioxidant biomarkers and their return to normal levels might refer to the adaptation of camels to CIDR by modulating their oxidant and antioxidant levels.

## Introduction

The one-humped camel (*Camelus dromedarius*) is an important animal in Africa and Asia having tremendous potential for milk, meat, and transport [1]. It is predicted that domestic camelids (camels, llamas, and alpacas) will be increasingly important for animal production in harsh environments due to their adaptive characteristics to desert [2]. However, dromedary camels are regarded as seasonal breeders with a relatively short breeding season during the cooler months [3]. Elevation of the ambient temperature during summer seems to play the main role in affecting the camel reproductive activities through disturbance of the physiological activities [4]. In Egypt, the breeding season has been reported to be from December to May [5], during spring [6], and from December to August [7]. Synchronization of follicular waves is important for the development of fixed time artificial insemination and embryo transfer programs [8,9]. In camels, multiple approaches to synchronize follicular wave have been adapted from other species but met with variable results [2]. Moreover, there are conflicting reports on the efficacy of progesterone (P_4_)-releasing intravaginal devices (PRIDs) [10] and controlled internal drug releases (CIDRs) [7] in synchronization of follicular waves in camels. While camels treated for 17 days with PRIDs containing 1.9 g P_4_ and receiving a large dose of equine chorionic gonadotropin (eCG) (3000 IU) had a better synchrony of follicular growth during breeding season [11], treatment with CIDRs containing 1.38 g of P_4_ for 10 days did not synchronize follicular waves in the non-breeding season [7]. On the contrary, estrus was induced in camels during the non-breeding season using eCG [12]. As well as, ovarian activity was also induced in Indian camels during the non-breeding season [13].

Reactive oxygen species (ROS) and antioxidant biomarkers have been detected in follicular fluid of dromedaries [14]. Oxidative stress occurs when the overproduction of the ROS could not be balanced by the production of antioxidants. This overproduction of ROS without sufficient antioxidant capacity results in oxidative damage that may cause many diseases including infertility [15]. Malondialdehyde (MDA) is one of the most frequently used indicators of lipid peroxidation [16] and it is a potential biomarker for oxidative stress as a biomarker of lipid peroxidation [17,18]. During various reproductive conditions, antioxidant such as superoxide dismutase (SOD) is naturally produced to prevent some disorders resulting from the overproduction of free radicals arising through oxidative phosphorylation and oxidase-catalyzed reactions [15,19]. In addition, glutathione-S-transferase (GST) plays a role in the biosynthesis of prostaglandins, T, and P_4_ [20]. Nitric oxide (NO) is a short-lived inorganic free radical gas whose effects and the mechanism of action of NO depend strictly on its concentration, and consequently, NO may exert dual effects on the same process in the same cell [21,22]. NO is hardly measurable with direct methods because it is unstable gas having a very short half-life. However, it is rapidly converted into nitrites and nitrates which can be measured in biological fluids [23].

Considerable development and research were accomplished in the area of estrus synchronization and controlled breeding in ruminants. However, it is not the case in camels [24]. Studies on biochemical parameters are important for clinicians in the field, especially during programs utilizing progestin sources such as synchronization programs, artificial insemination, and embryo transfer [25]. This study evaluated the effect of exogenous P_4_ hormone using CIDR for 7 days on blood concentrations of steroid hormones and oxidative status of dromedary she-camels during peak and low breeding seasons.

## Materials and Methods

### Ethical approval

The animals during the experiment were handled in accordance with the use and animal care committee of Desert Research Center, Egypt.

### Animal and management conditions

The experiment was conducted at Mersa-Matrouh Research Station, Desert Research Center, Egypt. A total of ten healthy one-humped, parous, non-pregnant camels of 9-13 years old (*Camelus dromedarius*) weighing 400-500 kg housed in semi-shaded pens under natural daylight and temperature were used. Animals were dewormed and vaccinated regularly. Camels were fed as one group on maintenance ration with concentrate mixture at the rate of 3-5 kg/head/day in addition to Egyptian clover hay (*Trifolium alexandrinum*) and water was presented once daily.

### Experimental design

During the breeding (March 2012) and non-breeding season (June 2012) [5], camels were treated with an EAZI-Breed CIDR insert containing 1.38 g P_4_ (Hamilton, New Zealand) for 7 days [26]. The minimum and maximum temperatures in summer (June-August) were 22°C-35°C with 56.0% mean relative humidity (RH) and in spring (March-May) 15°C-28°C with 48.7% RH. No male camel was existed nearby females to avoid the induction of ovulation by introducing the male.

### Ultrasound examination

SonoAce R2 Ultrasound Scanner (Medison, Samsung, South Korea), equipped with 12 MHz linear array B-mode transducer, was used to examine the ovaries to assure the reproductive status of she-camel before application of CIDR.

### Blood sampling

The blood samples were collected through jugular venipuncture each other day from the day following CIDR insertion (day 1) until day 5 after CIDR removal and then were allowed to clot, and after centrifugation at 3000 rpm for 15 min, sera were harvested and kept at −20°C until measuring hormones, oxidants, and antioxidants.

### Measured parameters

#### P_4_

Quantitative P_4_ in serum was assayed using Enzyme immunoassay kits (Legal Manufacturer, DRG Instruments, GmbH, Germany) according to the method of Katt *et al*. [27]. The sensitivity was 0.05 ng/ml, and intra- and inter-assay precisions were 5.9% and 10.1% for P_4_, respectively.

#### Estradiol (E_2_)

Quantitative assay of serum E_2_ [28] using Enzyme immunoassay kits (Legal Manufacturer, DRG Instruments, GmbH, Germany) was performed. The minimum detectable value was 2.0 pg/mL and the test precisions were 6.81% and 7.25% for E_2_.

#### Testosterone

Quantitative *in vitro* measurement of testosterone in serum according to Tietz [29] was performed using Enzyme immunoassay kits (Legal Manufacturer, DRG Instruments, GmbH, Germany). The sensitivity of the assay was 0.083 ng/mL, and the inter- and intra-run precision coefficients of variation were 4.16 and 9.94% for testosterone, respectively.

#### MDA

MDA level was determined colorimetrically according to the method of Ohkawa *et al*. [30] using kits purchased from Biodiagnostic, Egypt.

#### NO

Endogenous nitrite concentration (NO^2−^) as indicator of NO production in biological fluids was measured using Nitrite Assay Kit provided by Biodiagnostic, Egypt, according to Montgomery and Dymock [31].

#### SOD

SOD activity was estimated kinetically using SOD Assay Kits (Biodiagnostic, Egypt) according to Nishikimi *et al*. [32].

#### GST

GST activity was estimated kinetically using GST Assay Kits (Biodiagnostic, Egypt) according to Habig and Jakoby [33].

### Statistical analysis

Data were presented as means±standard error of the mean. Statistical analyses of the data were performed using SPSS^®^ software (SPSS Inc., Version 16, 2007, USA). Data were subjected to independent sample t-test to determine the effect of season (in and out). Simple one-way ANOVA was used to study the effect of the day within each season. Duncan’s multiple range test was used to differentiate between significant means at p<0.05. Univariate General Linear Model (2 seasons * 7 days) was processed for every parameter, and tests between subjects’ effects of treatment in and out season, days, and their interaction (days * treatment) were performed using Repeated Measures Analysis.

## Results

### Effect of exogenous P_4_ treatment during breeding season and non-breeding seasons on (P_4_) concentrations (ng/ml) in serum of dromedary she-camel in Egypt

**Figure-1:**
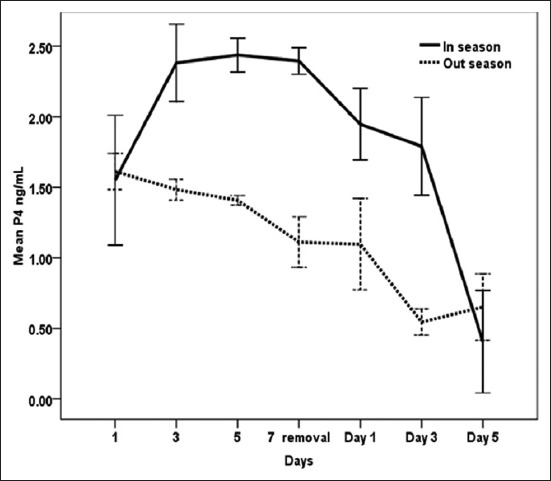
Effect of exogenous progesterone treatment during breeding season and non-breeding seasons on progesterone concentration (ng/ml) in serum of dromedary she-camel in Egypt.

Figure-1 shows that P_4_ serum levels changed significantly (p<0.05) during different days of CIDR application in she-camels during breeding and non-breeding seasons. There was significant (p<0.02) interaction between CIDR treatment and time (Table 1). During the breeding season, a rapid increase in serum P_4_ concentration was observed on day 1 after CIDR insertion (1.55±0.18). P_4_ concentrations reached maximum values (2.43±0.12) 5 days after CIDR insertion, maintained high levels until day 7, and then reached low values on day 5 after CIDR removal (0.41±0.03). During the non-breeding season, P_4_ increased 24 h after CIDR insertion (1.61±0.12), then descended gradually, and reached low values on day 3 (0.54±0.04) and day 5 (0.65±0.02) after CIDR removal. As shown in Table-1, the overall means of blood P_4_ concentration were significantly (p<0.05) lower during the non-breeding season in comparison with breeding season.

**Table-1:** Effect of breeding season on P_4_ (ng/ml), E_2_ (pg/ml), T (ng/ml), MDA (nmol/ml), NO (mmol/L), SOD (U/ml), and GST in dromedary she-camels treated with CIDR for 7 days.

Breeding season	Parameters						

	P_4_ (ng/ml)	E_2_ (pg/ml)	Testosterone (ng/ml)	MDA (nmol/ml)	NO (µMOL/L)	SOD (U/ml)	GST (µMOL/ml)
Peak breeding season	1.97^b^±0.14	53.66^b^±2.46	1.05^a^±0.06	7.13^b^±0.19	25.16^a^±0.32	310^a^±9.25	4103^a^±190
Low breeding season	1.15^a^±0.09	45.86^a^±3.17	1.55^b^±0.04	5.31^a^±0.15	26.43^a^±0.29	321^a^±10.52	5616^b^±490
Season (p-value)	0.01	0.014	0.01	0.01	0.009	0.217	0.01
Season*Time (p-value)	0.02	0.015	0.01	0.03	0.042	0.038	0.029

Data are presented as means±SEM. Means having different superscripts in the same column (a,b) differ significantly between breeding and non-breeding seasons. GST=Glutathione-S-transferase, CIDR=Controlled internal drug release, MDA=Malondialdehyde, SOD=Superoxide dismutase, NO=Nitric oxide, P_4_=Progesterone, E_2_=Estradiol, SEM=Standard error of the mean

### Effect of exogenous P_4_ treatment during breeding season and non-breeding seasons on E_2_ levels (pg/ml) in serum of dromedary she-camel in Egypt

**Figure-2:**
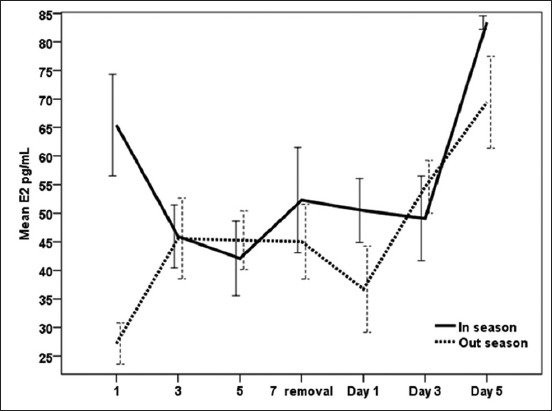
Effect of exogenous progesterone treatment during breeding season and non-breeding seasons on estradiol levels (pg/ml) in serum of dromedary she-camel in Egypt.

During both the seasons, CIDR treatment as well as interaction of time and treatment had a significant (p<0.05) effect on E_2_ (Figure-2 and Table-1). During the breeding season, E_2_ concentrations declined from the day following CIDR insertion (65.42±8.88) and then reached significantly (p<0.05) low values (15.70±0.09) on day 3 after CIDR removal and a significantly (p<0.05) high level on day 5 (83.40±1.17). During the non-breeding season, E_2_ concentrations showed the highest value on the day of CIDR removal (69.43±8.08), whereas the lowest value was noticed one day following CIDR insertion (27.19±3.59). It is clear from Table-1 that the overall means of blood E_2_ concentration were significantly (p<0.05) lower during CIDR in non-breeding season in comparison with breeding season.

### Effect of exogenous P_4_ treatment during breeding season and non-breeding seasons on testosterone concentrations (ng/ml) in serum of dromedary she-camel in Egypt

**Figure-3:**
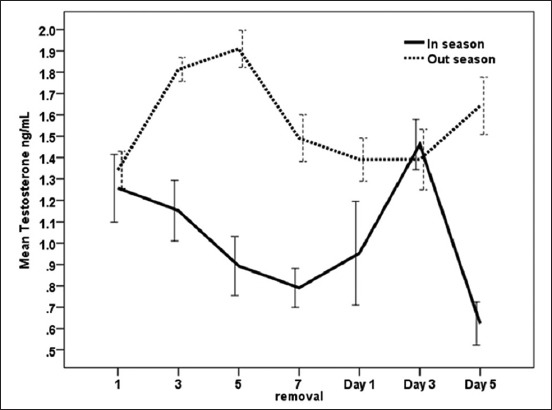
Effect of exogenous progesterone treatment during breeding season and non-breeding seasons on testosterone concentrations (ng/ml) in serum of dromedary she-camel in Egypt.

As shown in Figure-3, testosterone gradually decreased during the breeding season till reach low values on the day of removing CIDR (0.33±0.04), then increased till reach high value on day 3 (1.46±0.13) after removing it. During the non-breeding season, testosterone concentrations showed a significant increase on days 3 (1.81±0.06) and 5 (1.90±0.09) following CIDR insertion compared to the low values one day following CIDR insertion (1.34±0.09). Moreover, there were significant (p<0.05) increases in overall means of blood testosterone during non-breeding season than breeding season (Table-1). It is clear from Table-1 that there was significant (p<0.01) interaction between exogenous P_4_ treatment and days of CIDR.

### Effect of exogenous P_4_ treatment during breeding season and non-breeding seasons on MDA concentrations (nmol/ml) in serum of dromedary she-camel in Egypt

**Figure-4:**
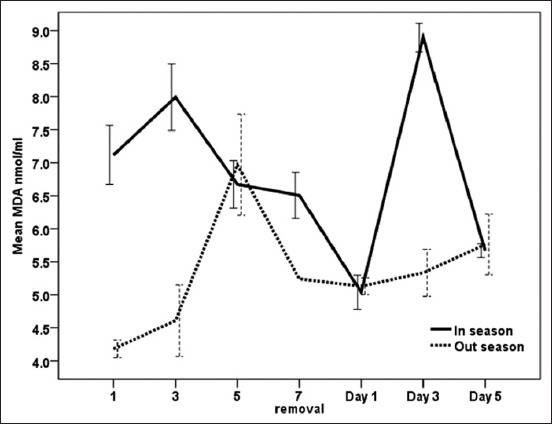
Effect of exogenous progesterone treatment during breeding season and non-breeding season on malondialdehyde concentration (nmol/ml) in serum of dromedary she-camel in Egypt.

During the breeding season and non-breading seasons, days during treatment had a significant (p<0.05) effect on MDA (Figure-4). During the breeding season, MDA reached significant (p<0.05) value on day 3 after CIDR insertion (7.99±0.50) and day 3 (8.89±0.22) after CIDR removal. During the non-breeding season, MDA increased significantly (p<0.05) reaching high value on day 5 (6.97±0.77) following CIDR insertion, and after CIDR withdrawal, a reincrease was observed on day 5 (5.76±0.46). Moreover, serum MDA was altered significantly (p<0.03) by interaction between days of CIDR and season (Table-1). It is also evident from values shown in Table-1 that MDA (p<0.05) was significantly lower during non-breeding season than high breeding season.

### Effect of exogenous P_4_ treatment during breeding season and non-breeding seasons on NO concentrations (µMOL/L) in serum of dromedary she-camel in Egypt

**Figure-5:**
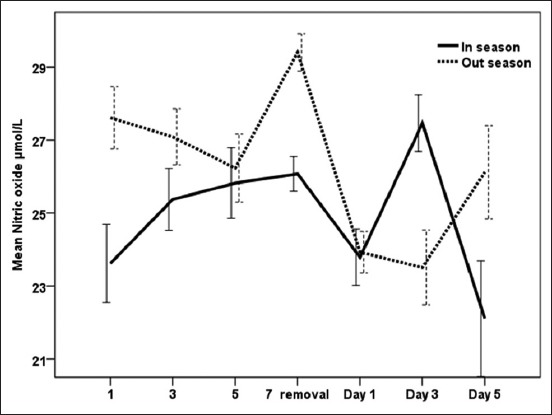
Effect of exogenous progesterone treatment during breeding season and non-breeding season on nitric oxide concentration (µmol/L) in serum of dromedary she-camel in Egypt.

Figure-5 clarifies that days during treatment in both the seasons had a significant (p<0.05) effect on NO which showed two peaks on day 7 on CIDR removal (26.07±0.11) and day 3 after CIDR removal (27.46±0.78). NO reached maximum values after CIDR insertion on day 1 (27.61±0.85) and day 7 (29.40±0.51). After CIDR withdrawal, however, NO decreased significantly (p<0.05) on day 3 (23.51±1.03) then reached another peak on day 5 (26.12±1.28; Figure-5). However, no significant difference (p<0.05) was found in NO levels between high and low breeding seasons (Table-1).

### Effect of exogenous P_4_ treatment during breeding season and non-breeding seasons on SOD concentrations (U/ml) in serum of dromedary she-camel in Egypt

**Figure-6:**
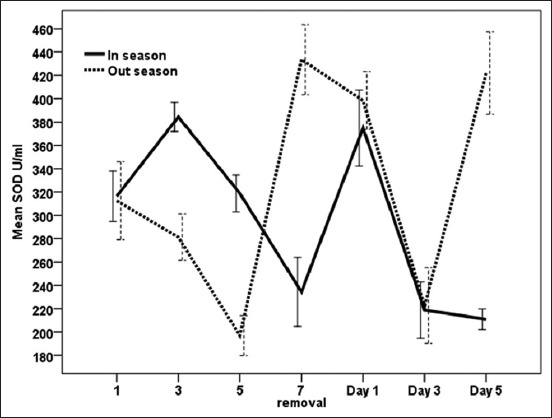
Effect of exogenous progesterone treatment during breeding season and non-breeding seasons on superoxide dismutase concentration (U/ml) in serum of dromedary she-camel in Egypt.

As shown in Figure-6, exogenous P_4_ treatment using CIDR in the breeding and non-breading seasons had a significant (p<0.05) effect on SOD. In addition, an interaction (p<0.03) between treatment and days of CIDR was recorded as presented in Table-1. During the breeding season, SOD increased significantly and reached maximum values on day 3 followed by a significant decrease of SOD activity until the day of CIDR removal (334±29) and rose again on day 1 after CIDR removal. During the non-breeding season, a significant increase of SOD activity was observed on day 7 when CIDR was removed (434±230) and day 5 after its withdrawal (422±35) compared to the decrease of its activity on day 5 (197±17) following CIDR insertion and day 3 (223±32) after its withdrawal. However, no significant difference (p<0.05) was found in SOD levels between high and low breeding seasons (Table-1).

### Effect of exogenous P_4_ treatment during breeding season and non-breeding seasons on GST concentrations (µMOL/ml) in serum of dromedary she-camel in Egypt

**Figure-7:**
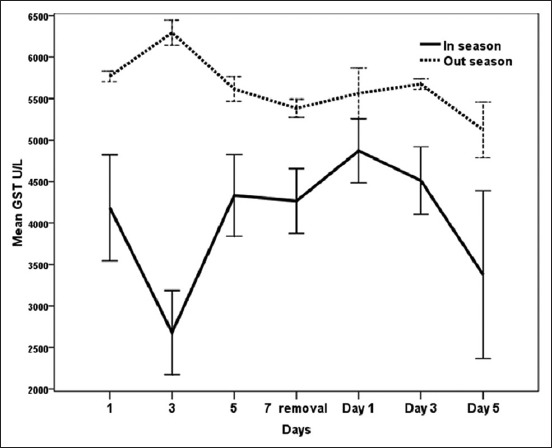
Effect of exogenous progesterone treatment during breeding season and non-breeding seasons on glutathione-S-transferase concentration (µmol/ml) in serum of dromedary she-camel in Egypt.

It is evident from Figure-7 that GST values during breeding season were significantly (p<0.05) lower on day 3 (2680±506) before removing CIDR in comparison with its high values on days 1 (4871±385) and 3 (4511±407) after CIDR removal. In the non-breeding season, GST levels showed peak values on day 3 (6294±152) during CIDR and descended to minimum values on day 7 of CIDR removal (5382±109) and day 5 (5122±336) after CIDR removal (Figure-7). Meanwhile, GST was significantly (p<0.05) higher during the non-breeding season than breeding season (Table-1).

## Discussion

In this study, P_4_ concentration was higher during peak breeding season when compared to non-breeding season. Moreover, synchronized she-camels exhibited a typical P_4_ profile during treatment days in the breeding season, as levels of P_4_ increased during CIDR insertion and declined at CIDR removal and thereafter. Conversely, E_2_ and testosterone concentrations decreased post-insertion of the CIDR and remained low until the day of removal, when they started to increase again. Similar to our dromedaries, llama treated with CIDR for 16 days showed a rapid increase in plasma P_4_ concentrations after the insertion of the intravaginal devices which reached peak values 1 day after insertion [34]. Moreover, there were interactions between treatment and days on serum levels of P_4_ and E_2_ as well as testosterone measured in the current work. These results are in partial agreement with those of Abd-El Hamid [35] who found a significant effect of interaction between treatment and days on levels of serum P_4_ in camels received CIDR (1.38 g) device for 10 consecutive days. On the other hand, the later author reported no interactions between days (0, 2, 4, 6, 8, 10, 15, 17, and 20) and treatments on serum concentrations of E_2_ in synchronized camels. According to Khalifa [36], the increase of P_4_ in peripheral circulation after CIDR insertion in she-camels is a result of effective absorption from vagina and removal of device would result in fast decline of the hormone in an expected way follow a biexponential curve. Skidmore *et al*. [25] reported that exogenous P_4_ could hasten the regression of large follicles, but it does not completely inhibit follicular growth in camels. However, in South American Camelids, Vaughan [37] found that 100 or 200 mg of P_4_, administered IM every 2 days, was effective in inducing regression of the existing dominant follicle and preventing new wave emergence, whereas Chaves *et al*. [34], by evaluating follicular diameters and sexual hormones concentrations during treatment, stated that CIDR (0.33 g) could be effective in completely preventing follicular development for a period of up to 7 days. Swelum and Alowaimer [38] observed fall in P_4_ levels and emergence of a new follicular wave after 2-4 days after CIDR withdrawal during the breeding season of camels in Egypt. According to Monaco *et al*. [7], P_4_ administration through CIDR (1.38 g) for 10 days during beginning and in the middle of the non-breeding season of camels in Egypt was inefficient for synchronizing follicular waves. Nevertheless, the treatment did not affect mean follicular number, demonstrating its failure in controlling the emergence of new follicular waves. Female camels are seasonal breeders affected by long daylight during the warmest months of the year [39]. Based on the historical records over a period of 12 years (1999-2010), summer temperatures are extremely high in Egypt reaching 38°C-43°C. Cairo subtropical climate is characterized by hot summer season extending from June to August with average minimum and maximum temperatures from 23°C to 35°C and 74% mean temperature humidity index [40]. The higher P_4_ levels during breeding season observed in the present study are in agreement with Babiker *et al*. [41] who reported an increase of P_4_ levels in female camels as an indicator of breeding season in dromedary camels in Sudan. On the other hand, Ali *et al*. [42] found higher serum P_4_ level during low than the peak breeding season in camels. The main source of serum E_2_ is the Graafian follicles [42], and regression of the follicles, on the other hand, was followed by low estrogen and testosterone concentrations [43]. In this study, a dose of 1.38 g P_4_ for 7 days for synchronization of estrus in she-camels induced significant decreases in E_2_ and testosterone blood levels during breeding season with higher serum E_2_ concentrations during the peak than the low breeding season. The changes in serum E_2_ levels in the present study could be attributed to the fact that ovarian growth of new follicles could be inhibited by progestin treatments [44]. The similar pattern of E_2_ and testosterone in dromedaries of this study and the association between the increase of E_2_ and testosterone concentrations after the removal of CIDR during both the seasons indicate their importance during follicle growth and maturation [45]. Plasma concentrations of testosterone were found to follow the same variations as peripheral concentrations of estrogen and increased size of the follicle is accompanied by an increase in peripheral concentrations of testosterone [46]. These results of E_2_ in the current experiment are consistent with the findings of Hozyen *et al*. [47] who recorded a significant decline in E_2_ after insertion of CIDR in dromedary she-camels for 10 days. The decrease in E_2_ after CIDR insertion during the breeding season was also observed in llama treated with CIDR for 16 days, where E_2_ concentrations decreased drastically by day 1 post-insertion of the CIDR and remained low until day 6, when E_2_ started to increase again [34]. Both E_2_ profiles and dominant follicle development attained a wave-like pattern in dromedaries and *Lama guanicoe*, showing a close relationship to follicle size [46,48]. On the other hand, Swelum and Alowaimer [38] revealed a non-significant difference in E_2_ levels during and after CIDR treatment in dromedary camels. In the non-breeding season, E_2_ remained low or showed irregular, small increases due to incomplete waves of follicular development [46]. Similar to our results, higher plasma E_2_ concentrations were observed during peak breeding than low breeding season in camels [49]. Higher frequency of animals with active ovaries during the peak (86.76%) than the low (45.83%) breeding season were was in camel, and consequently, ovaries had more active follicles during the peak breeding season, resulting in higher serum E_2_ levels than the low breeding season [42].

Camels are well adapted to their environment by modulating their redox defense mechanisms against ROS and oxidative stress [50]. Ayres *et al*. [51] reported that lipid peroxidation is most often induced by O^−2^ and SOD catalyzes the reaction that converts O^−2^ to H_2_O_2_ and molecular oxygen. Furthermore, a number of studies have been conducted to measure NO levels to analyze oxidative stress. The obtained results in this work revealed that interactions between treatment and time significantly (p<0.05) affected measured oxidant and antioxidant biomarkers. There was a significant increase of MDA on day 3 after CIDR insertion and after CIDR removal during the breeding season and on the days 3 and 5 during CIDR in the non-breeding season. Moreover, NO levels increased on the day of CIDR removal during both the seasons and SOD activities were high on day 3 during CIDR and 1 day after CIDR removal during the breeding and the non-breeding seasons. On the other hand, GST decreased after CIDR insertion in both the seasons. This is in agreement with Oral *et al*. [52] who reported that PRID caused stress to the animals, and MDA and NO concentrations rose after device application procedures in healthy heifers and then returned to normal levels. In the same respect, the pattern of MDA during CIDR was also observed when CIDR was used in cows [53]. Increases in MDA levels could be attributed to vaginitis and stress that occurs after CIDR application and removal [53]. Moreover, the enzymatic antioxidant GSTs are capable of detoxifying several ROS produced during different physiological processes [20]. In the current work, different patterns of oxidant/antioxidant biomarkers were observed when comparing breeding and non-breeding seasons in camels treated with exogenous P_4_ for 7 days. As MDA in serum increased significantly during breeding season, SOD and GSTs were significantly higher during the non-breeding season when compared to breeding season. Increased oxidants such as MDA and NO in goats were related to increased serum P_4_ concentration after administration of intravaginal P_4_-releasing devices for estrus synchronization [54,55]. Furthermore, short-term PRID treatments increase serum P_4_ levels but decrease total antioxidant capacity in dairy heifers [23]. Elevated P_4_ and decreased E_2_ levels during breeding season in the present study were associated with increased NO and decreased SOD activities. The increase of NO on the day of CIDR removal during the breeding and the non-breeding seasons in the present work could confirm the association of low level of NO with the ovarian activity and follicle growth [15]. According to Gragasin *et al*. [56], estrogen improved the bioavailability of NO by modulating the function and expression of endothelial NO synthase.

## Conclusion

From the present study, it could be concluded that: (1) The increase in P_4_ and decrease in E_2_ after CIDR insertion during the breeding season and the inverse pattern for these hormones during the non-breeding season could indicate that dose 1.38 g P_4_ for 7 days was efficient during the breeding season and not during the non-breeding season in Egyptian dromedary she-camels. (2) Alterations in concentrations of blood oxidant and antioxidant biomarkers after CIDR insertion and its return to normal levels refer to the adaptation of camels to CIDR by modulating their oxidants and antioxidants levels.

## Authors’ Contributions

RHM, AME, HFH, and MAM designed the experiment. HFH, AHA, and AMAE performed the analyses of all measured parameters. RHM, AME, and MAM provided the animals and carried out the experiment. AHA contributed to statistical analysis. HFH and AMAE drafted the manuscript. RHM, AME, MAM, and AHA helped to draft the manuscript. HFH and RHM reviewed the manuscript efficiently. All authors read and approved the final manuscript.
